# Recycling of a Concrete Pavement after over 80 Years in Service

**DOI:** 10.3390/ma13102262

**Published:** 2020-05-14

**Authors:** Tomasz Rudnicki, Robert Jurczak

**Affiliations:** 1 Faculty of Civil Engineering and Geodesy, Military University of Technology in Warsaw, 2 Gen. S. Kaliskiego St., 01-476 Warsaw, Poland; 2 Department of Road and Bridge Engineering, West Pomeranian University of Technology in Szczecin, 50 Piastow St., 70-311 Szczecin, Poland; robert.jurczak@zut.edu.pl; 3 Department in Szczecin, General Directorate for National Roads and Motorways, 33 Bohaterów Warszawy St., 70-340 Szczecin, Poland

**Keywords:** recycled concrete aggregates, concrete pavement, recycling, air void analysis in hardened concrete, durability

## Abstract

This article presents the results of fatigue testing and assessment of the mechanical and physical properties of the concrete pavement of the A6 motorway, which was put in service in 1938. After 82 years of operation under heavy traffic loading conditions, the pavement was fully recycled by crushing of the existing concrete and reuse of the reclaimed material in the new courses of pavement placed as part of the motorway renewal project. The main objective of this research was to determine the properties of the tested concrete, including compressive strength, water absorption and freeze-thaw resistance after 150 cycles of alternate freezing and thawing. The resistance of the concrete to the action of de-icing products was also checked. The article also presents the results of petrographic analysis of the aggregates. Additionally, concrete sampled from the pavement was evaluated for freeze-thaw resistance in relation to the determined porosity characteristics. The tested concrete, which was subjected to over 80 years of traffic loading on the A6 motorway, was found to meet the highest requirements as currently applied for the extra heavy-duty pavements. With a compressive strength value in excess of 50 MPa, the tested concrete can be rated at least CC40, according to EN 13877-2:2013-08. The samples were found to satisfy the freeze-thaw resistance requirements of an F150 rating. The air void analysis showed that the analyzed concrete contained 1.6% of micropores, i.e., air voids smaller than 300 μm (A_300_). The spacing factor, in turn, was below 0.200 mm (*L* = 0.185 mm). The example of the A6 motorway renewal project served to demonstrate that reclaimed concrete aggregate, obtained by crushing the entire pavement, can be used for production of the new pavement courses.

## 1. Introduction

Recycling offers several ways to utilize the qualities of old concrete. As one of the available options, recycled concrete materials can be used for production of new concrete [1,2,3]. So far, this option has been generally limited to using the reclaimed crushed aggregate in the new concrete mix in place of some amounts of fine and coarse natural aggregates. The properties of such concrete, which depend on the origin and on the proportion of recycled aggregate, are generally inferior to the properties of concrete containing only natural aggregate. Although the cutting edge recycling technologies are impressively efficient in separating the aggregate from the cement matrix, there are barriers to their wider application, including a high cost involved in the processing of recycled aggregate and problems with utilization of the dust generated as a by-product of the process [4]. Use of recycled concrete pavement aggregate as the only material for construction of the lower courses of pavement (road base, stabilized subgrade) appears to be an option of choice from both economic and technological standpoints. The idea to use the recycled concrete pavement aggregate for road base layers has been successfully implemented in several places worldwide, including the United States, China and Norway [5,6,7]. Notably, the percentage of concrete pavements in the overall road network in Poland is small, namely ca. 5%. The renewal methods used on these sections of the Polish road network include rubblising or black topping techniques [8]. The results of other research projects [9,10,11] show a possibility to use recycled pavement aggregate for the production of sub-base course and cement-treated subgrade materials. The application of aggregate obtained as a result of crushing the old concrete pavement of the A6 highway at the demolition site (using commonly used crushing equipment) to build in and reuse that aggregate as the pavement layers allows the reduction of its production costs to a minimum.

In comparison to other modern concrete recycling methods (e.g., the heating and rubbing method or mechanical grinding method), this mainly avoids the additional costs associated with separating the aggregate from the adjacent hardened cement slurry (paste). In addition, there is no problem in the management of small fractions of concrete debris that are a by-product of these technologies and contain a significant amount of hardened cement slurry (paste).

The objective of this research is to evaluate the properties of the over 80-year-old concrete pavement and to confirm the possibility of reusing the recycled material as a quality aggregate for new built or renewal projects. The in-place recycling of the concrete pavement to obtain a quality aggregate brings major environmental benefits, including reduced extraction of natural aggregates and reduction of CO_2_ emission and, besides, reduces the cost of the road construction works. In the authors’ opinion, the material obtained from the analyzed over 80-year-old pavement features very good strength properties, which are accompanied by an adequate freeze-thaw resistance and homogeneity.

The analyzed section of the concrete pavement between the interchanges Szczecin Dąbie and Rzęśnica was built in 1938 as part of the Reichsautobahn (RAB) No. 4 motorway expansion scheme of the pre-war Germany, which was planned to connect, in the final layout, Berlin in the west with Gdańsk in the east, reaching up to Kaliningrad (Königsberg). Currently, it is part of the A6 motorway on the European route E28, linking the north-western part of the Polish road network with the German road network. In addition, it serves also as the southern by-pass of Szczecin. On this section, the A6 route coincides with the route of the S3 expressway (trunk road) heading to Świnoujście, Poland. It is worth mentioning that the operated section of the A6 motorway is at present the only concrete paved road section among the national highways in the Western Pomerania region of Poland.

**Figure 1:**
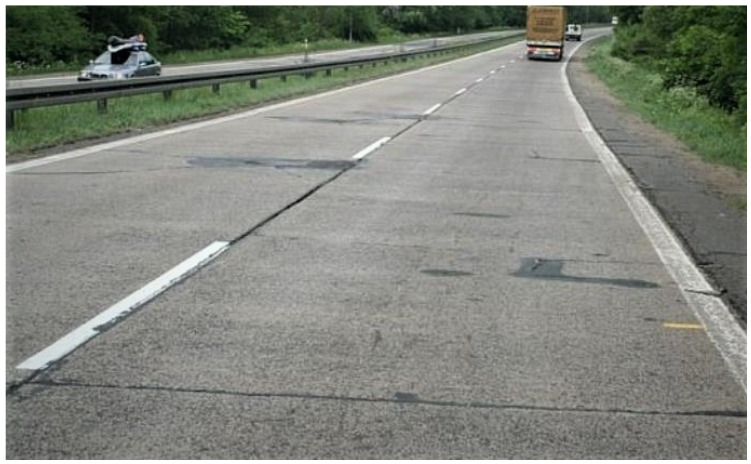
View of the A6 motorway before renewal.

During the over 80 years of operation no renewals were carried out on the analyzed section. Up to the early 1990s hardly any repairs were done either. Later on, the scope of maintenance was limited to making good the distressed concrete slabs, including transverse and longitudinal cracks. The condition of the motorway before the renewal project is shown in the picture below (Figure 1).

**Figure 2:**
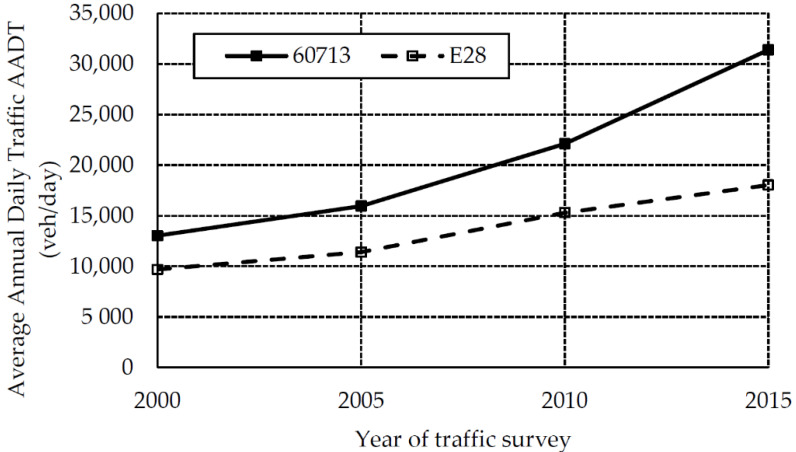
The data of the 2000–2015 national traffic survey (GPR) for the analyzed section of the A6 motorway between the interchanges Rzęśnica and Szczecin Dąbie (traffic count station No. 60713).

The cause of cracks and other distress was the specific composition and the high volume of traffic of as much as 31,384 vehicles/day, as counted at the traffic count station No. 60713 during the survey in 2015. A point must be made that the volume of traffic has been increasing over many years. For example, in the period 2000–2015 it increased on the analyzed section by 140% (Figure 2). In 2015 the average annual daily traffic on the A6 motorway was almost two times higher than on the European route E28. According to the 2015 traffic count data, the percentage of heavy vehicles, which have a decisive bearing on the progress of pavement deterioration, was ca. 15%.

The assessment of the concrete pavement was related to the A6 renewal project with the planned full recycling of the pavement structure on the section under analysis.

## 2. Materials and Methods

### 2.1. Materials

**Figure 3:**
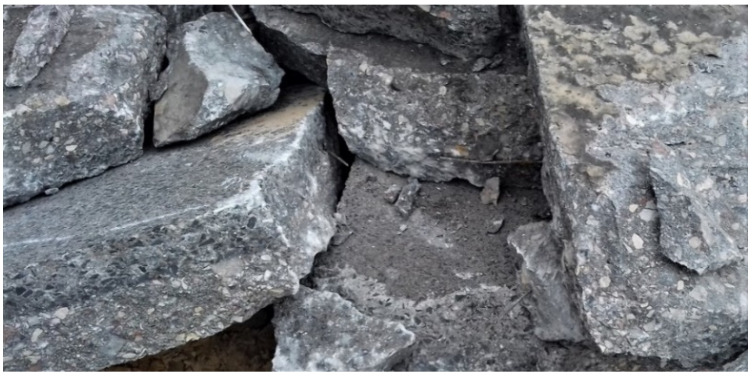
View of broken concrete pavement from which the test samples were cut.

As part of this research, cores were cut from the existing concrete pavement in order to assess the main fatigue life parameters (Figure 3). In addition, larger pieces of the pavement were also taken during the site preliminaries on the project section of the motorway, which were subsequently used to prepare the specimens for laboratory testing to determine the porosity characteristics and for the petrographic analysis.

### 2.2. Methods

As part of condition assessment of the concrete pavement the test specimens were prepared for tests carried out to:

— Obtain a simplified petrographic description according to EN 932-3:1999/A1:2004 [12],
— Determine the compressive strength of concrete according to EN 12390-3:2019 [13],
— Determine the density of concrete according to EN 12390-7:2011 [14],
— Determine the water absorption of concrete according to the Polish Standard No. PN-B-06250:1988 [15],
— Determine the F150 freeze-thaw resistance of concrete according to the Polish Standard No. PN-B-06250 [15],
— Determine the porosity characteristics in hardened concrete according to EN 480-11:2008 [16],
— Determine the freeze-thaw resistance with de-icing salts according to EN 1340:2004/AC:2007 [17].

Then, in order to assess the suitability of aggregate obtained from the concrete recycling, its physical and mechanical properties were determined by carrying out basic laboratory tests, which covered the following scope:

— Determination of resistance to fragmentation by the Los Angeles test method according to EN 1097-2:2010 [18],
— Determination of water absorption of analysed aggregates according to EN 1097-6:2013-11 [19],
— Determination of aggregate resistance to cycling action of freezing and thawing according to EN 1367-1:2007 [20],
— Determination of California bearing ratio (CBR) according to 13286-47:2012 [21].

In practice, the properties listed above decide on the acceptance or rejection of material for use in individual layers of the pavement structure and the reinforced (improved) soil.

The article also presents the results of laboratory tests carried out by the GDDKiA’s road testing laboratory of Olsztyn (Olsztyn, Poland) on the concrete cores cut from the pavement of the S22 expressway during the renewal of the section between the city of Elbląg, Poland and the state border of Poland. The method and the time of construction of this pavement were the same as of the pavement of the analyzed section of the A6 motorway. The compressive strength, freeze-thaw resistance and water absorption tests were carried out, plus determinations of tensile splitting strength according to EN 12390-6:2019 [22], water permeability according to PN-B-06250:1988 and water penetration depth according to EN 12390-8:2011 [23].

Visual examinations of the cores cut from the A6 motorway showed that the concrete pavement of ca. 21 cm total thickness was placed in two lifts applied with the wet-on-wet method (Table 1). The upper layer of over 7 cm in thickness (7.0 to 8.5 cm) was made of a concrete mix containing coarse basalt aggregate. The lower layer, of 12.5 cm to 14.0 cm in thickness (13 cm on average) was, in turn, made of a concrete mix containing uncrushed aggregate. This was a typical method of construction of concrete pavements at that time [24]. The results of a detailed petrographic examination are presented in 3.1 below. The concrete slabs were placed directly on mechanically compacted subgrade without a sub-base layer in between. The native soil subgrade was built of fine sand.

**Figure 4:**
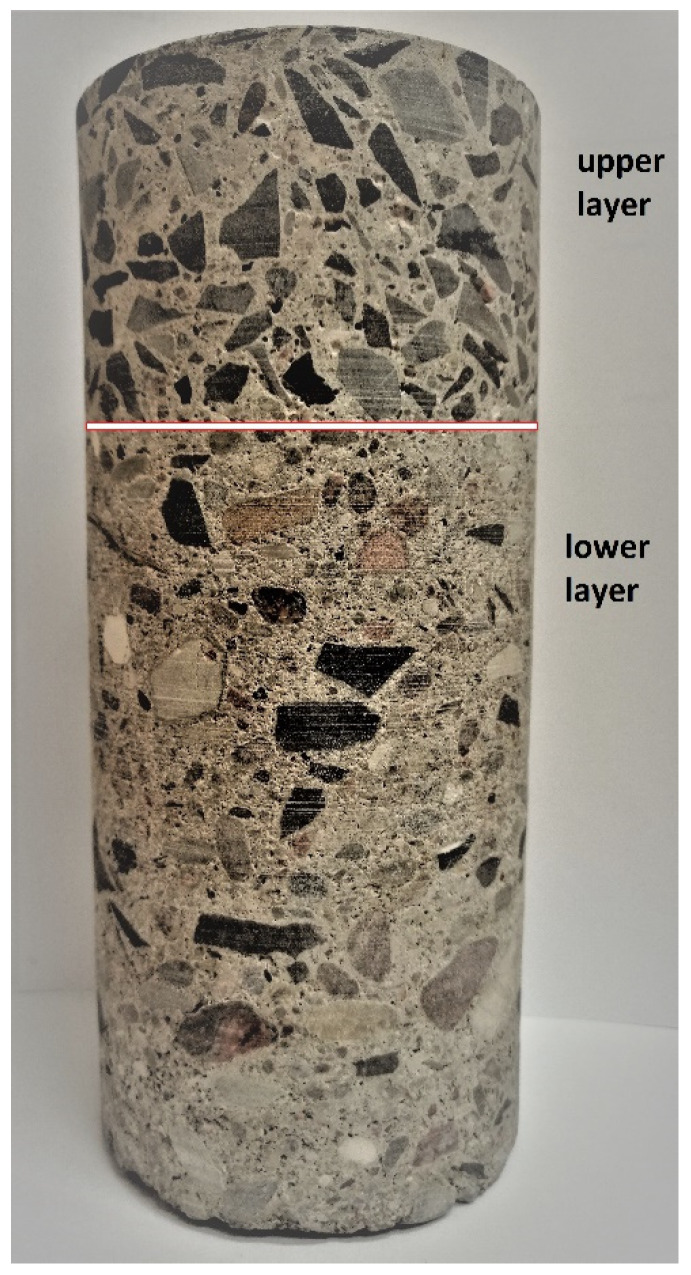
View of the core cut from the A6 pavement.

In the core shown in the picture below (Figure 4) one can distinguish the upper and lower layers.

All the cores cut from the A6 pavement were conditioned in accordance with the standard procedure and polished when used for measurement of the mechanical properties.

## 3. Results and Discussion

### 3.1. Petrographic Examination of Aggregate according to EN 932-3:1999/A1:2004 

**Figure 5:**
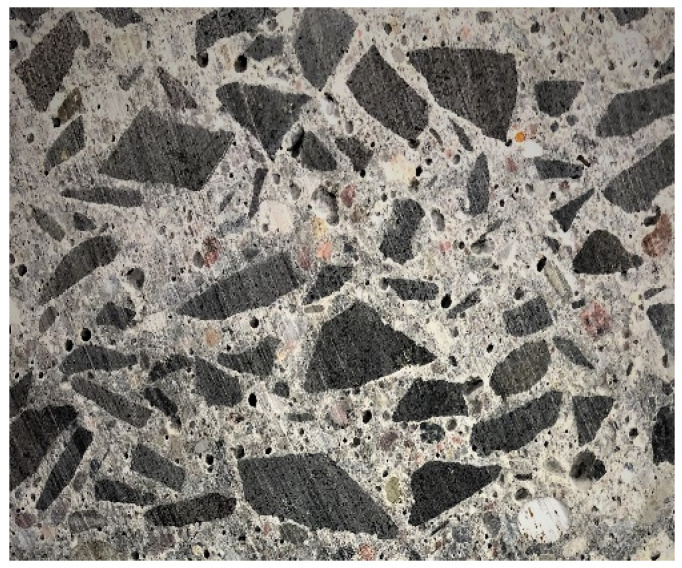
Petrography of the upper layer.

The main component of the upper layer mix was a crushed igneous rock, namely basalt [25] (basaltoide), black in colour, 4/22 in size. This rock features a porphyritic structure and massive, non-directional texture. The microscopic evaluation revealed a fully porphyritic structure with numerous olivine (magnesium) phenocrystals and infrequent monoclinic salite pyroxenes. Besides igneous aggregate the cement paste contains ca. 40% of light colour quartz grains (Figure 5).

**Figure 6:**
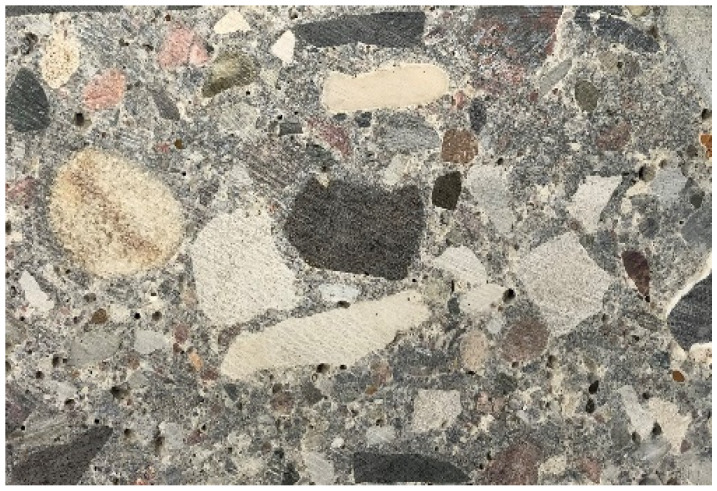
Petrography of the lower layer.

In the lower layer (Figure 6), the main component is a sedimentary material, namely gravel of 4/20 fraction, with 85% to 92% grains of magmatic and metamorphic origin. The remaining 8–15% of grains come from sedimentary rocks (mainly limestone).

### 3.2. Hardened Concrete Properties

#### 3.2.1. Density

The density of concrete was determined on the total number of six cores cut from the pavement, three from each of the two layers. The test procedure of EN 12390-7:2011 [14] was used and the obtained densities were 2515 kg/m^3^ for the upper layer and 2404 kg/m^3^ for the lower layer. All the test results are compiled in Table 2.

#### 3.2.2. Compressive Strength

The compressive strength of concrete was determined on the total number of six cores, three from each of the two layers. The test procedure of EN 12390-3:2019 [13] was used and the results were 65.8 MPa for the upper layer and 51.2 MPa for the lower layer. All the test results are compiled in Table 3.

The strength of the upper layer concrete was higher than the strength of the lower layer concrete (by ca. 28%). The strength test procedure according to EN 13791:2008 [28] was applied as for the cores cut from the pavement structure. The characteristic compressive strength of the tested concrete, corresponding to the strength determined on 150 mm test cubes, depends on the number of results and their variation. For a smaller number of results (k = 7) and small variation the value of characteristic strength can be taken at up to 58.8 MPa for the upper layer and up to 44.2 MPa for the lower layer. This gives the strength classes of C55/67 and C40/50 for the upper and lower layers, respectively. If assessed according to EN 13877-2:2013-08, these classes change to CC55 and CC40 for the upper and lower layers, respectively.

Considering the recommendations [29] regarding reuse of concrete pavement materials, derived as part of the research project named RID I/6: *Reuse of Recycled Materials*, it needs to be pointed out that in terms of the compressive strength the concrete obtained from recycling of the A6 motorway pavement satisfies the requirements specified for concrete that after crushing could be used for unbound sub-base and treated subgrade layers and also for road base layer construction, i.e., it can be rated in classes CC20 and CC30 respectively.

#### 3.2.3. Water Absorption

Absorption, defined as an increase of weight of concrete due to uptake of water, was determined according to the Polish Standard No. PN-B-06250:1988 [15] on a total number of six cores, three from each of the two layers of the pavement in consideration. First, the samples were dried at 105 °C until their weight no longer decreased, and then, after weighing, they were immersed in water and weighed when fully saturated with water. The absorption value of ca. 3.5% was obtained for both the tested concrete types. The test results are compiled in Table 4 below.

It is worthwhile to note that the obtained absorption values are much lower than the limit of less than 5% as specified for concrete exposed to direct environmental actions according to PN-B-06250:1988 [15].

#### 3.2.4. Determination of Freeze-Thaw Resistance

EN 206:2014-04 [26] does not provide for experimental verification of the freeze-thaw resistance of concrete. Thus, the freeze-thaw resistance of concrete is determined on the basis of the minimum strength class, the minimum cement content, the maximum w/c ratio and the minimum air void content, as specified for a given exposure class, checked with the test procedures and the rating criteria given in the specifications used by the Polish highway agency GDDKiA. In this research the freeze-thaw resistance of concrete was determined according the Polish Standard No. PN-B-06250:1988 [15]. The tests were carried out on the total number of six samples, three from each of the two layers (designated *g*—for the upper layer and *d—*for the lower layer), which following the tests were subjected to 150 freeze-thaw cycles while the remaining samples were kept immersed in water. After 150 cycles the samples were subjected to visual examination. The samples showed neither surface distress nor cracking. Next, the samples were weighed and subjected to the compressive strength test. The test results are compiled below in Table 5.

From these results it transpires that after 150 freeze-thaw cycles at the test temperatures of −20 °C for freezing and +20 °C for thawing the tested concrete featured very good fatigue resistance, which allows us to conclude that the exposure to heavy traffic and de-icing salts used as winter maintenance agents (the motorway runs through an area with frequent daily freeze-thaw events) had not caused deterioration of the pavement. The average loss of weight in the tested samples was 0.15%. The decrease of compressive strength was less than 20%, viz. 16.2% for the upper layer concrete and 6.1% for the lower layer concrete. These values satisfy the freeze-thaw resistance requirement.

#### 3.2.5. Determination of Resistance to Ice Melt (De-Icing) Salts

Resistance to the action of de-icing products was determined with the method used for the rating of precast concrete units, such as curbs and pavers. The freeze-thaw resistance in the presence of de-icing salts was tested according to the procedure of EN 1340:2004/AC:2007 [17] concrete curbs – requirements and test methods, on three samples cut from the upper layer of the A6 motorway, which were prepared according to the test procedure as described below. In the first step, a rubber seal was placed on each sample projecting 2 cm above the tested surface and then the perimeter chamfers were filled with a silicone sealant to close the gaps between the concrete and the rubber seal. Next, the side and bottom surfaces were insulated with an insulating foam and EPS. For fifteen minutes before placement in the freezing chamber, the deionized water layer was removed from the sample and replaced with a 5 mm high layer of 3% water solution of NaCl. Next the samples were subjected to alternate freezing and thawing cycles. The weight of scaled material was determined after 28 and 56 cycles. The results obtained in this test are presented in Table 6 below.

In each case the mass loss from a single sample was below 1.5 kg/m^2^. The mean mass loss after 28 and 56 freeze-thaw cycles was below 1.0 kg/m^2^. The obtained values of freeze-thaw resistance in the presence of de-icing salts satisfy the requirements of FT2 freeze-thaw category, as defined in EN 13877-2:2013-08 [27].

### 3.3. Parameters of Concrete Sampled from the Road Pavement on the Section of S22 Road Between Elbląg and the State Border of Poland.

The same kind of testing was simultaneously carried out at the GDDKiA’s road testing laboratory of Olsztyn for the concrete pavement of the Elbląg-Kaliningrad road constructed in 1938 using the cores obtained during renewal of the section between the city of Elbląg, Poland and the state border of Poland. The tests were carried out on nine 100 mm cores and three 150 mm cores. The test results are compiled in Table 7 below.

The compressive strength, absorption and freeze-thaw resistance data obtained for the concrete of the S22 pavement are very close to the values obtained for the A6 pavement. In addition, the tests carried out at the GDDKiA’s road testing laboratory of Olsztyn revealed a very tight structure of the tested concrete, as confirmed by low water penetration. The tensile splitting strength values satisfy the requirements of the assumed tensile splitting strength class SC 5.0 according to EN 13877-2:2013-08 [27].

### 3.4. Air Void Analysis of Hardened Concrete

The air void size distribution was determined according to EN 480-11:2008 [16]. The analysis was carried out on specially prepared 150 mm × 100 mm × 20 mm metallurgical polished sections in the laboratory of the Military University of Technology in Warsaw. Four such sections were used in the tests, which, after cleaning and drying, were polished and coated with a contrast agent. The following air void system parameters were determined for each of the analyzed samples of hardened concrete:

— Total air content *A*%,
— Specific surface of the air void system *α* mm^−1^,
— Spacing factor *L* mm,
— Micro air void content (amount of air voids below 0.3 mm in size) *A_300_*%,
— Paste-air ratio *R*%.

**Figure 7:**
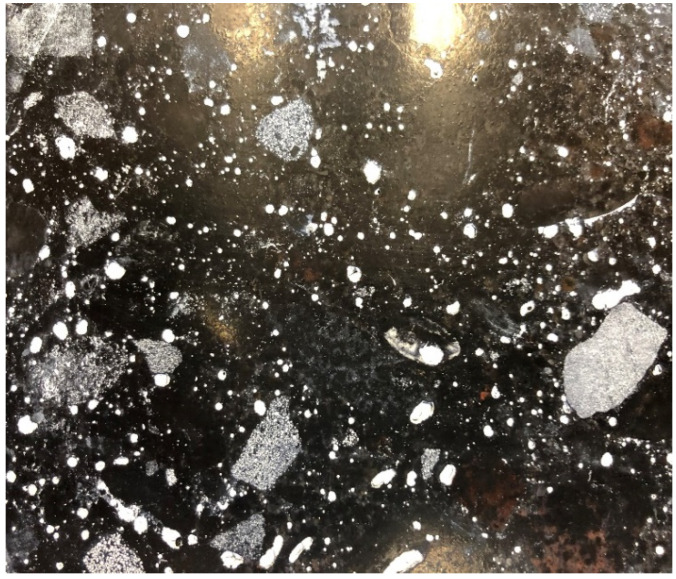
Sample No. 1.1. ready for metallurgic examination.

**Figure 8:**
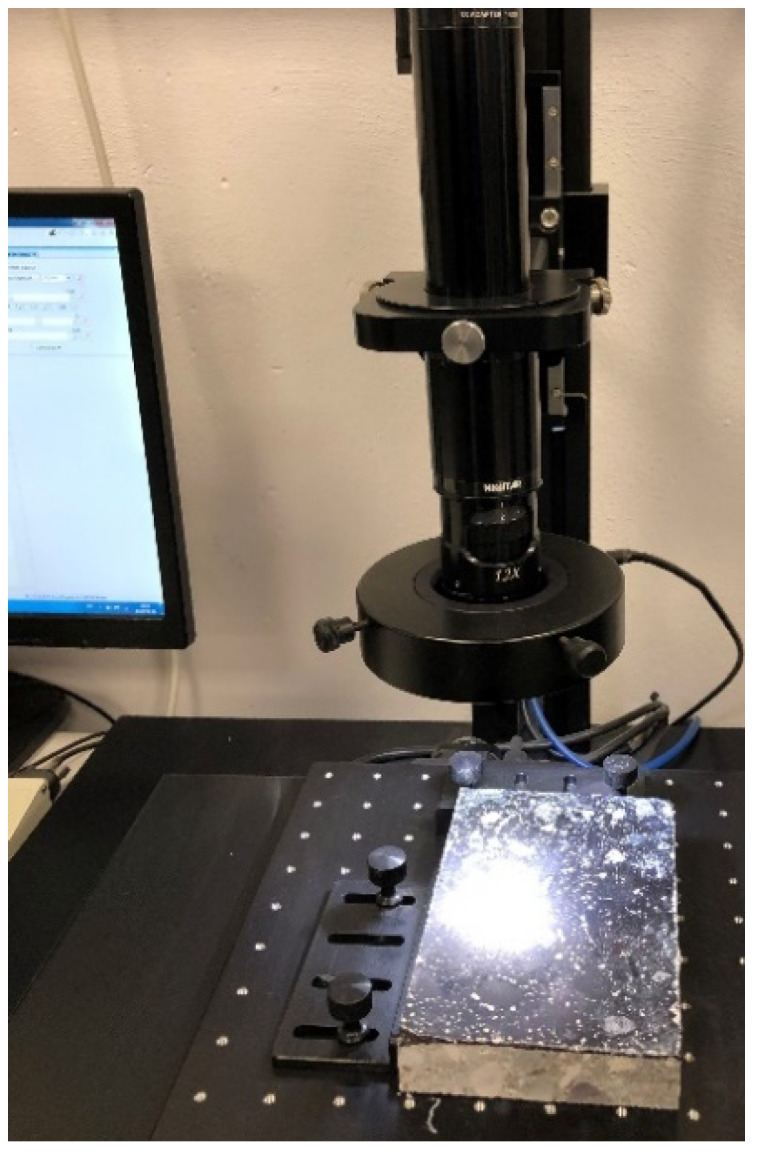
Sample No. 1.1. during metallurgic examination.

A computer image analysis method was used to evaluate the microstructure of concretes and the parameters of pore structure were calculated; these parameters included relative volume fraction, relative specific surface area, and pore arrangement ratios [30]. The samples after polishing with already applied contrast agent are presented in Figure 7 and Figure 8.

The results of metallurgic examination of the four samples are presented in Table 8.

**Figure 9:**
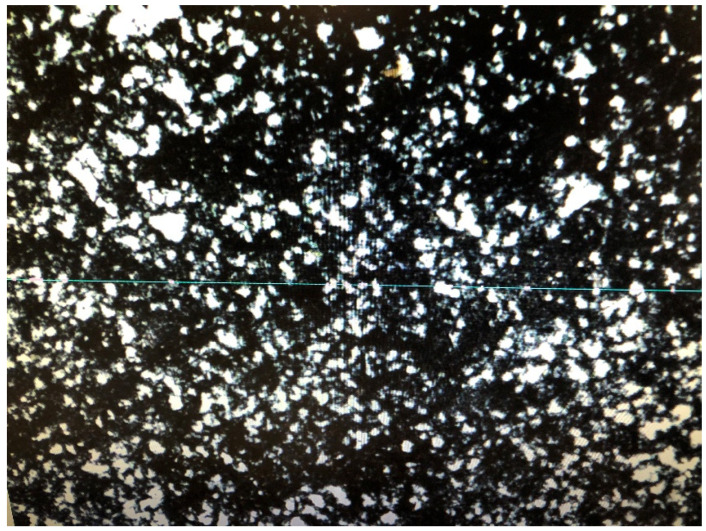
Air void size distribution in sample No. 1.1 during examination.

An image of sample 1.1 taken from the lower layer of pavement during examination is shown in Figure 9.

**Figure 10:**
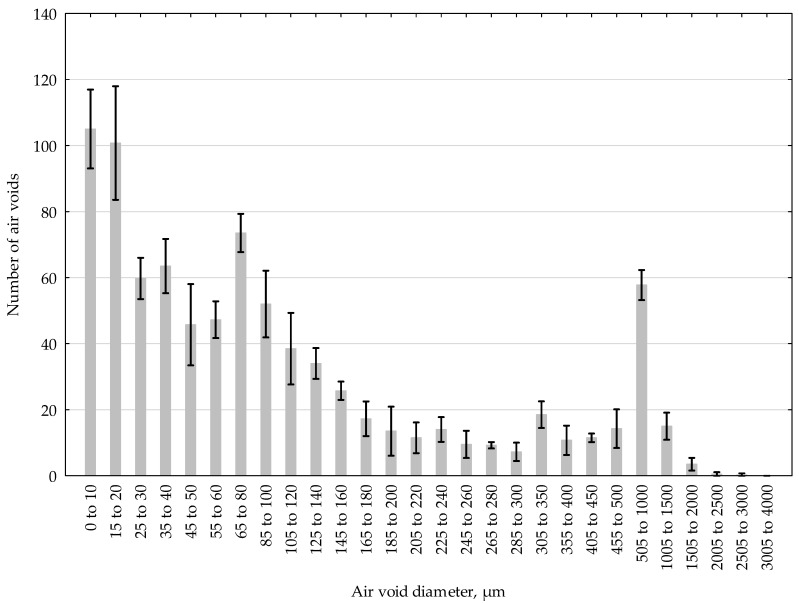
Air void size distribution determined during examination.

The air void size distributions determined during examination of samples are presented in Table 9 below and are presented graphically in Figure 10.

Figure 10 shows the mean value with standard deviation. For determining the air void size distribution, a specific calculation model was adopted in which certain pore diameters are defined [31]. This model provides a middle-of-the road representation between the actual situation and the Powers model, the latter of which assumes equal size of air voids [32,33]. The chords are measured and classified into one of the pre-defined length ranges, and then the number of chords in a given range is multiplied by a volume of a single air void of a diameter equal to the upper limit of the range. In this way, the predicted amount of air is obtained for a given size range.

The air void distribution parameters determined according to EN 480-11:2008 [16] in the laboratory of the Military University of Technology in Warsaw allow us to conclude that the tested, over 80-year-old concrete satisfies very high requirements as currently specified for paving grade concrete. The total amount of air *A* ranged from 5.4% to 6.1%, the specific surface α ranged from 23.1 to 26.0 mm^−1^ and the paste/air ratio *R* ranged from 4.2 to 4.8. Most important for the freeze-thaw resistance evaluation are, however, the values of the spacing factor *L* and the micro-air content *A_300_*, which were in the ranges of 0.17 to 0.20 mm and 1.51–1.86% respectively.

### 3.5. Recycling of Concrete Pavement

**Figure 11:**
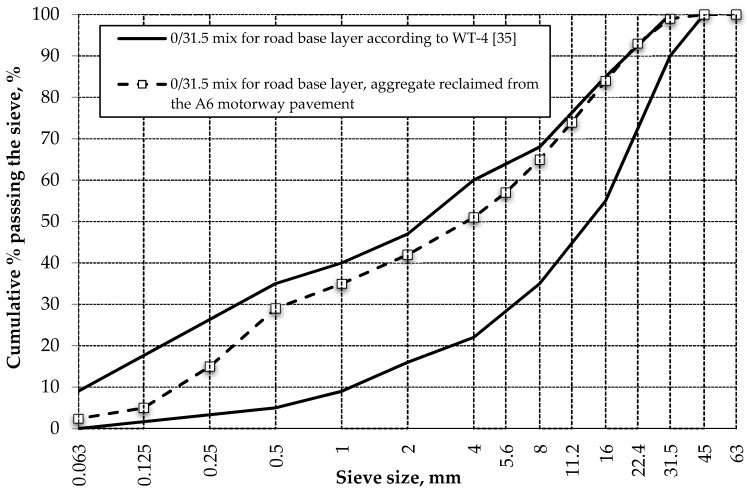
Example grading curve of 0/31.5 mm continuously graded reclaimed aggregate from a recycled concrete pavement (derived on the basis of [36]).

The concrete slabs of the A6 motorway pavement, which were planned to be recycled to provide reclaimed aggregate, featured very good physical and mechanical properties, as confirmed by the test results presented in 3.2 and 3.3 above. In the authors’ opinion, the above-described reclaimed material can, following appropriate processing, be used to substitute some of the new aggregate in concrete mixes for paving applications, even for the lower layers of pavement. Unfortunately, the Polish guidelines and specification do not provide for the use of aggregate reclaimed from concrete pavements for production of new PCC pavement mixes. Therefore, it was decided to use all the reclaimed material for construction of the road base layer, the foundation of the new pavement. The concrete pavement crushing process produced a 0/31.5 mm continuously graded material which, in relation to the on-going A6 renewal project, was subjected to the essential quality control tests at the GDDKiA’s road testing laboratory of Szczecin, Poland. The tested parameters included grain size distribution according to EN 933-1:2012 [34] and CBR according to EN 13286-47:2012 [21]. These results were compared with the domestic requirements (called as WT-4 for short) of [35]. An example grading curve of mechanically crushed reclaimed concrete aggregate is presented in Figure 11 below.

The grading curve of the 0/31.5 mm mixture containing reclaimed aggregate obtained from recycled pavement slabs of the A6 motorway fell within the grading envelope. The amount of particles smaller than 0.063 mm was 3% (m/m), which is less than the maximum allowed. The tested 0/31.5 mm mixture, compacted in a laboratory at optimum moisture content, featured a very high value of CBR (ca. 160%) [37]. The requirements of [32,35] specify the CBR limits defining suitability of the material for strengthening of subgrade, sub-base and road base construction of 40%, 60% and 80%, respectively.

The crushing value, absorption and freeze-thaw resistance of the reclaimed concrete aggregate were also determined (Table 10). According to the test data, the reclaimed aggregate fails to satisfy the absorption requirement. This requirement can, however, be dropped in the case of reclaimed aggregates which satisfy the freeze-thaw resistance requirement (mass loss not greater than 10%). The freeze-thaw test data, based on which the reclaimed material is classified in the F_4_ category, confirm the suitability of this material for the construction of the road base and sub-base layers and for subgrade treatment as well. A very good crushing value (LA_35_) of the reclaimed material is also worthwhile noting.

### 3.6. Discussion

The tests indicated very good physical and mechanical properties of the concrete under analysis. The strength properties satisfy at least the requirements for class C40/50 according to EN 206:2014-04 [26] or CC40 according to EN 13877-2:2013-08 [27]. The high quality of the tested concrete was confirmed by the obtained absorption values, all of which were below 4%. Considering the results obtained on the samples taken from the S22 pavement, constructed in the same way and at the same time as the A6 motorway pavement, one can reasonably expect that the concrete of the A6 pavement will also satisfy the requirements regarding the minimum tensile splitting strength SC2.0. The high strength parameters and long fatigue life of the pavement can be attributed to low fineness of cement [38] of grading close to 100 μm, which, in combination with the above-mentioned phase composition, resulted in a relatively small degree of hydration. This, at the same time, provided an excellent protection from the action of corrosive solutions owing to the self-healing effect of damaged micro-spaces. This small hydration [25,39] over such a long period of time also indicated a low w/c ratio of the mix [40]. The only chemical admixture that could have possibly been used at the time of construction are lignosulfonates [41,42,43], which are known for their strong set-retarding properties [44]. The interfacial transition zone is compact and indicates a very good bond between the cement paste and grains of aggregate, which, in combination with the very good quality of the latter, produces excellent strength and low permeability, the factors responsible for the exceptional durability of the analysed concrete pavement of the motorway.

The freeze-thaw resistance values after 150 cycles of alternate freezing and thawing testify to a very high quality and durability of the concrete in question. Furthermore, the experimentally determined freeze-thaw resistance in the presence of rock salt (NaCl) testify to its resistance also to the combined frost and de-icing salt exposure. The top layer concrete can be classified in the FT2 freeze-thaw resistance category. The air void analyses of the samples of the over 80-year-old concrete of the A6 motorway pavement showed that the concrete satisfies the contemporary requirements specified for PCC pavements, which confirms the long-lasting, perpetual character of pavements of this kind. The reclaimed aggregate obtained from crushing the entire pavement of the A6 motorway satisfies the requirements of [35] code of practice in terms of the crushing value and the freeze-thaw resistance. Hence it can be used for the road base layers and subgrade improvement works.

## 4. Conclusions

The following conclusions can be drawn on the basis of the authors’ testing and analyses of the test data:

(1) The test of compressive strength was 65.8 MPa for the upper layer and 51.2 MPa for the lower layer. With the compressive strength value in excess of 50 MPa, the tested concrete can be rated as at least CC40.
(2) The freeze-thaw resistance values after 150 cycles of alternate freezing and thawing testify to a very high quality and durability of the concrete in question.
(3) The air void analysis showed that the analyzed concrete contained 1.6% of micropores, i.e., air voids smaller than 300 μm (A_300_), the spacing factor, in turn, was below 0.200 mm (*L* = 0.185 mm). The air void analyses of the samples of the over 80-year-old concrete of the A6 motorway pavement showed that the concrete satisfies the contemporary requirements specified for PCC pavements, which confirms the long-lasting, perpetual character of pavements of this kind.
(4) The example of the A6 motorway renewal project served to demonstrate that reclaimed concrete aggregate, obtained by crushing the entire pavement, can be used for production of the new pavement courses.

Furthermore, the results of the tests carried out at the GDDKiA’s laboratory of Szczecin as part of the on-going quality control in relation to the A6 renewal project confirm satisfaction of the grading and CBR value requirements.

## Figures and Tables

**Table 1:** Thickness of the concrete pavement layers, as measured on the cores.

No.	1	2	3	4	5	6	7	8	9	10	11	12	13	14	15	16	Mean
Upper layer	74	75	81	80	83	71	85	72	83	70	74	80	75	80	70	75	77
Lower layer	130	128	130	130	125	130	130	130	135	140	130	130	135	130	135	125	131
Concrete pavement	204	203	211	210	208	201	215	202	218	210	204	210	210	210	205	200	208

**Table 2:** Densities of concrete.

No.	Upper Layer (kg/m^3^)	Lower Layer (kg/m^3^)	Mean Value (kg/m^3^)
1	2506	-	2515
2	2499		
3	2541		
4	-	2415	2404
5		2373	
6		2425	

**Table 3:** Concrete compressive strength test results.

No.	Compressive Strength-Upper Layer (MPa)	Compressive Strength-Lower Layer (MPa)	Mean Compressive Strength (MPa)	Compressive Strength Class of Concrete According to EN 206:2014-04 [26]	Compressive Strength Class of Concrete According to EN 13877-2:2013-08 [27]
1	66.5	-	65.8	C55/67	CC55
2	65.9				
3	65.1				
4	-	50.2	51.2	C40/50	CC40
5		49.0			
6		54.4			

**Table 4:** Water absorption values of the tested concretes.

No.	Water Absorption Upper Layer (%)	Water Absorption Lower Layer (%)	Mean Absorption Value (%)
1	3.3	-	3.4
2	3.4		
3	3.5		
4	-	3.4	3.5
5		3.5	
6		3.5	

**Table 5:** Experimentally determined freeze-thaw resistance of concrete.

No.	Rc (MPa)	Rc_150_ (MPa)	_Δ_R (%)	_Δ_M (%)
1 g	66.5	57.5	16.2	0.18
2 g	65.9	57.8		
3 g	65.1	50.2		
4 d	50.2	48.2	6.1	0.12
5 d	49.0	43.1		
6 d	54.4	53.0		

**Table 6:** Experimentally determined freeze-thaw resistance of concrete in the presence of de-icing salt.

No.	Mass Loss (kg/m^2^)		Mean Mass Loss (kg/m^2^)	
	After 28 Cycles	After 56 Cycles	After 28 Cycles	After 56 Cycles
1	0.13	0.21	0.09	0.65
2	0.11	0.43		
3	0.04	1.29		

**Table 7:** Results of tests on cores cut from the concrete pavement of the S22 expressway.

No.	Property		Unit	Result
1	compressive strength according to EN 12390-3:2019 [13]	Upper layer	MPa	62.9
		Lower layer	MPa	49.9
2	tensile splitting strength of the lower layer concrete, according to EN 12390-6:2019 [22]		MPa	5.0
3	absorption (teste don the entire core) according to PN-B-06250:1988 [15]		%	4.0
4	freeze-thaw resistance of the upper layer concrete according to PN-B-06250:1988 [15]	_Δ_R	%	3.8
		_Δ_M	%	0.6
5	water tightness of the upper layer concrete according to PN-B-06250:1988 [15]		mm	15
6	water tightness of the upper layer concrete according to EN 12390-8:2011 [23]		mm	10

**Table 8:** Experimentally determined air void system parameters of hardened concrete.

Parameter	Unit	Sample No. 1.1	Sample No. 1.2	Sample No. 2.1	Sample No. 2.2
Total traverse length, *T*	mm	2.464	2.464	2.464	2.464
Total air content, *A*	%	5.4	5.4	6.1	6.0
Total number of chords measured, *N*		861	877	939	863
Specific surface of the air void system, *α*	mm^−1^	26	23.3	24.9	23.2
Paste/ air ratio, *R*	%	4.8	4.8	4.2	4.3
Spacing factor, *L*	mm	0.18	0.20	0.17	0.19
Micro air void content, *A_300_*	%	1.64	1.57	1.86	1.51

**Table 9:** Size distribution and structure of air voids in the tested samples.

Size Range (µm)	Sample No. 1.1	Sample No. 1.2	Sample No. 2.1	Sample No. 2.2
	Number of Recorded Chords (voids)	Number of Recorded Chords (voids)	Number of Recorded Chords (voids)	Number of Recorded Chords (voids)
0 to 10	120	108	100	92
15 to 20	95	80	120	108
25 to 30	56	53	64	66
35 to 40	63	53	73	65
45 to 50	49	32	61	41
55 to 60	47	45	42	55
65 to 80	78	75	76	65
85 to 100	60	39	60	49
105 to 120	23	43	48	40
125 to 140	38	31	38	29
145 to 160	27	24	29	23
165 to 180	17	10	20	22
185 to 200	14	5	23	12
205 to 220	10	7	11	18
225 to 240	14	18	9	15
245 to 260	5	7	13	13
265 to 280	9	10	10	8
285 to 300	9	4	10	6
305 to 350	15	16	19	24
355 to 400	15	6	14	8
405 to 450	11	12	13	10
455 to 500	17	21	11	8
505 to 1000	57	59	52	63
1005 to 1500	9	16	17	18
1505 to 2000	2	2	6	4
2005 to 2500	1	0	0	1
2505 to 3000	0	1	0	0
3005 to 4000	0	0	0	0

**Table 10:** Compilation of the reclaimed concrete aggregate test data.

Property	Reference Standard	Unit	Aggregate Reclaimed from the A6 Motorway Pavement	Requirements acc. to WT-4 [35] Code of Practice	
				Subgrade and Sub-Base	Road Base
Aggregate crushing value 10/14	EN 1097-2:2010 [18]	%	31.8	LA_50_^1^	LA_40_^2^
Absorption 8/16	EN 1097-6:2013-11 [19]	%	3.8	WA_24_^2^	WA_24_^2^
Freeze-thaw resistance 8/16	EN 1367-1:2007 ^3^ [20]	%	3.5	F_10_	F_10_

^1^ Does not apply to subgrades improved according to WT-4 [35]; ^2^ LA_35_ for KR5 and higher traffic classes, as defined in WT-4 [35]; ^3^ Freeze-thaw resistance test is required only for aggregates with absorption value higher than 2%.
